# Assessing regional hepatic function changes after hypertrophy induction by radioembolisation: comparison of gadoxetic acid-enhanced MRI and ^99m^Tc-mebrofenin hepatobiliary scintigraphy

**DOI:** 10.1186/s41747-023-00409-x

**Published:** 2024-01-29

**Authors:** Matthias P. Fabritius, Benjamin Garlipp, Osman Öcal, Daniel Puhr-Westerheide, Holger Amthauer, Thomas Geyer, Adrien Holzgreve, Jens Ricke, Dennis Kupitz, Oliver S. Grosser, Jazan Omari, Maciej Pech, Max Seidensticker, Freba Grawe, Ricarda Seidensticker

**Affiliations:** 1 Department of Radiology, LMU University Hospital, LMU Munich, Munich, Germany; 2 General Surgery, Otto Von Guericke University, Magdeburg, Germany; 3 Department of Nuclear Medicine, Charité-Universitätsmedizin Berlin, Berlin, Germany; 4 Department of Nuclear Medicine, LMU University Hospital, LMU Munich, Munich, Germany; 5 Department of Radiology and Nuclear Medicine, University Hospital Magdeburg and Medical Faculty of Otto-Von-Guericke University, Magdeburg, Germany; 6 Research Campus STIMULATE, Otto-Von-Guericke University, Magdeburg, Germany

**Keywords:** Gadolinium ethoxybenzyl DTPA, Liver, Magnetic resonance imaging, Technetium Tc 99 m mebrofenin, Radioembolisation

## Abstract

**Background:**

To compare Gd-ethoxybenzyl diethylenetriamine pentaacetic acid (Gd-EOB-DTPA)-enhanced magnetic resonance imaging (MRI) and ^99m^Tc-labelled mebrofenin hepatobiliary scintigraphy (HBS) as imaging-based liver function tests after unilateral radioembolisation (RE) in patients with primary or secondary liver malignancies.

**Methods:**

Twenty-three patients with primary or secondary liver malignancies who underwent Gd-EOB-DTPA-enhanced MRI within a prospective study (REVoluTion) were evaluated. REVoluTion was a prospective open-label, non-randomised, therapy-optimising study of patients undergoing right-sided or sequential RE for contralateral liver hypertrophy at a single centre in Germany. MRI and hepatobiliary scintigraphy were performed before RE (baseline) and 6 weeks after (follow-up). This exploratory subanalysis compared liver enhancement on hepatobiliary phase MRI normalised to the spleen (liver-to-spleen ratio (LSR)) and the muscle (liver-to-muscle ratio (LMR)) with mebrofenin uptake on HBS for the total liver (TL) and separately for the right (RLL) and left liver lobe (LLL).

**Results:**

Mebrofenin uptake at baseline and follow-up each correlated significantly with LSR and LMR on MRI for TL (≤ 0.013) and RLL (≤ 0.049). Regarding the LLL, mebrofenin uptake correlated significantly with LMR (baseline, *p* = 0.013; follow-up, *p* = 0.004), whereas with LSR, a borderline significant correlation was only seen at follow-up (*p* = 0.051; *p* = 0.046).

**Conclusion:**

LSRs and LMR correlate with mebrofenin uptake in HBS. This study indicates that Gd-EOB-DTPA-enhanced MRI and ^99m^Tc-labelled mebrofenin HBS may equally be used to assess an increase in contralateral liver lobe function after right-sided RE.

**Relevance statement:**

MRI may be a convenient and reliable method for assessing the future liver remnant facilitating treatment planning and monitoring of patients after RE-induced hypertrophy induction.

**Key points:**

• Both MRI and HBS can assess liver function after RE.

• Liver enhancement on MRI correlates with mebrofenin uptake on HBS.

• MRI might be a convenient alternative for estimating future liver remnants after hypertrophy induction.

**Graphical Abstract:**

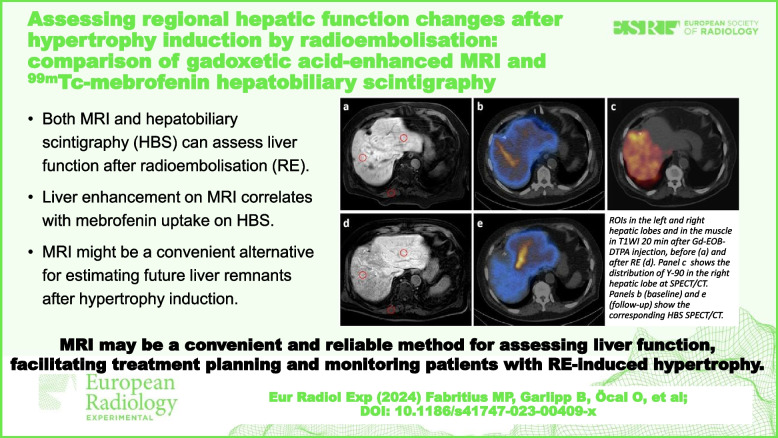

## Background

Liver surgery plays a vital role in treating liver malignancies. A significant number of patients lack an adequate future liver remnant (FLR) required for surgery, rendering them ineligible despite having resectable tumours [1]. FLR can be increased through a preoperative procedure to render them eligible, such as the established portal vein embolisation, portal vein ligation or associating liver partitioning with portal vein ligation in staged hepatectomy (ALPPS) [2–4]. Recent data indicate that unilateral radioembolisation (RE) is also effective in setting the stage for secondary operability in terms of hemihepatectomy with the advantage that it simultaneously represents a therapy of the tumour-affected liver [5–10]. Thus, in the case of aggressive tumours, the patient benefits from additional time while also indirectly acquiring predictive information regarding the potential benefits of secondary resection, acting as a “test of time”.

An adequate assessment of FLR after hypertrophy induction is crucial as posthepatectomy liver failure is the most dramatic complication following liver resection [11, 12]. Established methods include liver function testing and different clinical grading systems, such as the Child–Pugh score and the MELD score [13–15]. These methods are limited by the influence of extrahepatic factors on the assessments and the inability of attributing functional changes to specific liver regions [16]. The indocyanine green (ICG) clearance test quantifies the liver’s ability to eliminate ICG dye, often serving as a reference standard for global liver function evaluation [17–19]. However, this test relies on hepatic blood flow, which can be affected by factors like intrahepatic shunting and is unable to detect regional variations within the liver. Scintigraphic methods, mainly utilising tracers like ^99m^Tc-iminodiacetate analogues such as mebrofenin, have become increasingly established in recent years in clinical practice for evaluating tracer uptake and biliary excretion preoperatively [20–23]. Dynamic ^99m^Tc-mebrofenin hepatobiliary scintigraphy (HBS) or single-photon emission computed tomography (SPECT) can assess global and regional liver function and predict the FLR [24].

Besides scintigraphic methods, magnetic resonance imaging (MRI) and computed tomography (CT) volumetry are used to evaluate liver regional function with spatial information. However, the relationship between liver metabolic function and volume increase after hypertrophy-inducing procedures remained uncertain for some time [15, 25–30]. In the recent prospective REVoluTion study of 23 patients, it was shown that after unilobar RE, there is not only an increase in the volume of the contralateral lobe but also a significant increase in metabolic function as measured by HBS [7].

However, the assessment of liver function is not limited to these more complex methods but can also be estimated relatively easily by liver MRI with hepatocyte-specific contrast agents [19, 31]. MRI of the liver with gadoxetic acid (Gd-ethoxybenzyl diethylenetriamine pentaacetic acid (Gd-EOB-DTPA)) is a standard of care for detection, treatment planning, and follow-up of liver tumours. Several studies have already shown that conclusions about liver function can be drawn from the storage behaviour in the hepatocyte-specific phase, also after procedures like RE or radiation therapy [31–38]. Moreover, MRI measurements have been shown to correlate with HBS measurements, and the assessment of respectability after hypertrophy induction after portal vein embolisation seems possible [39–41]. However, it remains uncertain whether these findings can be extrapolated to the assessment of increased metabolic function FLR following one-sided RE.

Therefore, the objective of this post hoc analysis was to compare the utility of static gadoxetic-enhanced MRI and ^99m^Tc-mebrofenin HBS in evaluating changes in liver function after inducing hypertrophy by unilateral RE.

## Methods

### Study population

This post hoc analysis included all twenty-three patients of the prospective open-label, non-randomised, single-centre, therapy-optimising REVoluTion study [7]. The study was approved by the institutional ethics committee at Otto von Guericke University, Magdeburg (Ref. 08/14) according to the Declaration of Helsinki of 2013, and written informed consent was acquired from each patient. Patients underwent right-sided ^90^Y-RE for liver malignancies without underlying liver disease or biliary obstruction. They were aged 18–85 with an Eastern Cooperative Oncology Group (ECOG) performance status of 0–2. The exclusion criteria encompassed baseline non-embolised lobe functional volume > 30% of total functional liver volume, liver cirrhosis, pre-existing portal vein thrombosis, primary liver malignancies with cirrhosis or biliary dilatation, prior left liver lobe therapy/surgery or planned within 6 weeks of right-lobar RE, > 10% tumour involvement in left lobe, and contraindications for MRI, ^99m^Tc-mebrofenin HBS, or ICG test [7]. MRI and HBS were performed immediately before (baseline) and 6 weeks after RE (follow-up).

### MRI

**Fig. 1:**
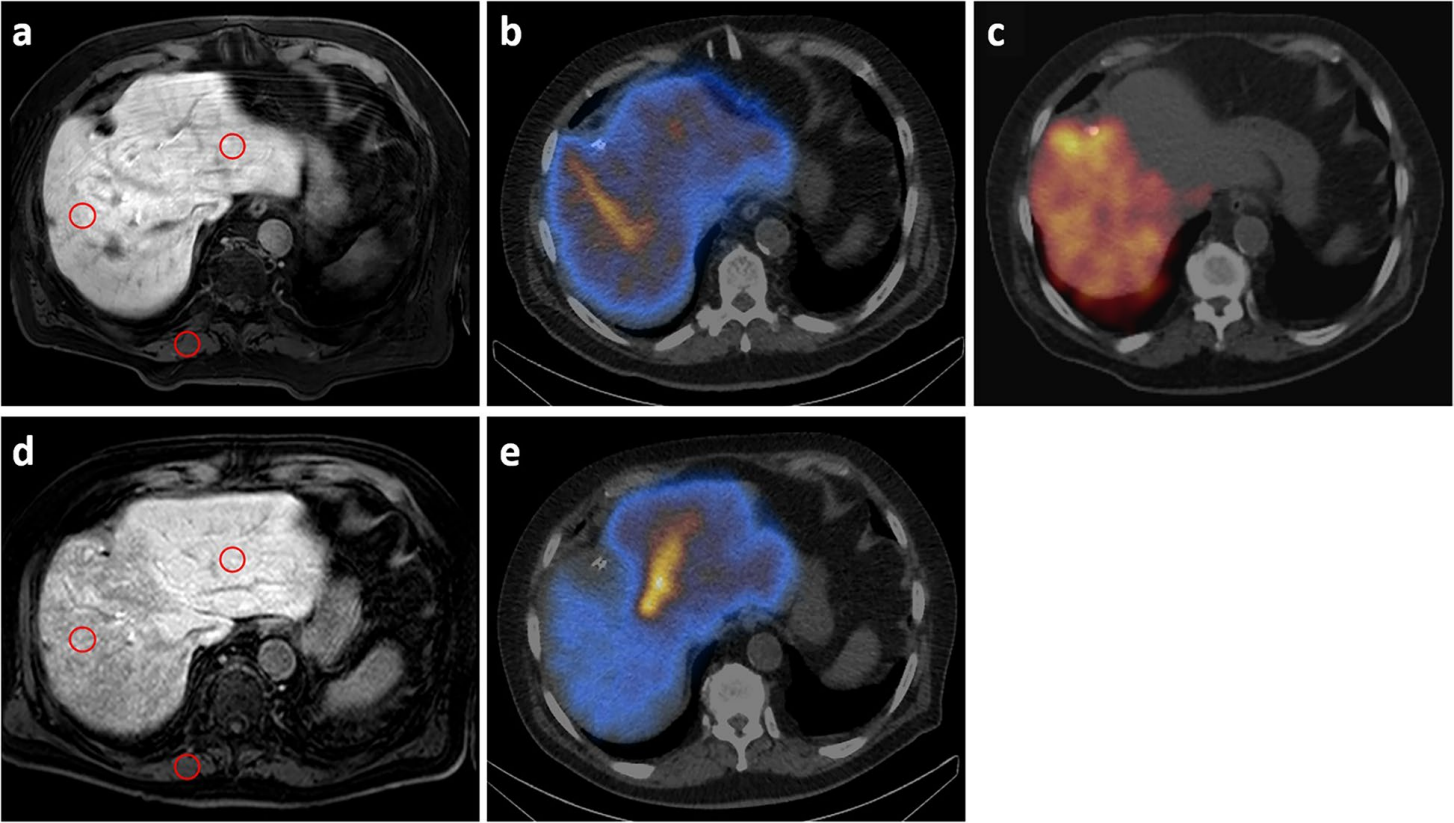
Case example of measurement of gadolinium ethoxy-benzyl diethylenetriamine pentaacetic acid (Gd-EOB-DTPA) uptake after radioembolisation. Regions of interest are placed in the left and right hepatic lobes (red circles), the spleen (not shown), and in the muscle in T1-weighted images obtained 20 min after Gd-EOB-DTPA, before (**a**) (baseline) and after RE (**d**) (follow-up). The measured mean signal intensities are used to calculate Gd-EOB-DTPA uptake according to Eqs. 1 and 2 (see the “Methods” section). The mean values at baseline (**a**) in RLL/LLL: LSR 1.9/1.9; LMR 1.8/1.8. The mean values measured at follow-up (**d**) in RLL/LLL: LSR 1.6/2.4; LMR 1.9/2.9. **c** The distribution of Y-90 microspheres in the right hepatic lobe in Bremsstrahlung SPECT/CT. **b** Baseline and **e** follow-up show the corresponding HBS SPECT/CT. HBS, Hepatobillary scintigraphy; LLL, Left liver lobe; LMR, Liver-to-muscle ratio; LSR, Liver-to-spleen ratio; RLL, Right liver lobe; SPECT/CT, Single-photon emission computed tomography/computed tomography

All patients underwent a standardised MRI protocol with 0.025 mmol/kg/body weight of Gd-EOB-DTPA (Primovist, Bayer Healthcare, Leverkusen, Germany,) on a 1.5-T scanner (Achieva, Philips Healthcare, Best, The Netherlands) using an 8-channel body-phased array coil. A standard axial T1-weighted high-resolution isotropic volume sequence, THRIVE (repetition time 4.0 ms, echo time 2.0 ms, and slice thickness 3 mm), was acquired 20 min after contrast injection (hepatobiliary phase) covering the entire liver. The contrast agent was manually injected in an anterior cubital vein, followed by a saline flush of 20 mL. Images were evaluated by a radiologist (M.S.) with over 15 years of experience in abdominal imaging using OsiriX v.8.0.2 (Pixmeo; Bernex, Switzerland). To prevent measurement bias, the radiologist was blinded to every other data. The mean signal intensity (SI) values of the liver, spleen, and paravertebral muscle were calculated using a manually placed circular region of interest (ROI) with a fixed size of 3 cm^2^, excluding major artefacts, liver lesions (including tumour), visible blood vessels, and biliary ducts. The mean SI of the liver was measured with four ROIs separately within the healthy parenchyma of each liver lobe (2 ROIs per liver lobe on different slices). One ROI each was located in the paravertebral muscle and spleen (Fig. 1). The mean SI was then calculated for the total liver (TL), the left liver lobe (LLL), and the right liver lobe (RLL). The liver-to-spleen (LSR) and the liver-to-muscle (LMR) ratios were calculated with the following formulas [34]:

$$\begin{array}{l}\begin{array}{cc}\mathrm{LSR }= \frac{{\mathrm{SI}}_{\mathrm{post}}\mathrm{\ of\ the\ liver}}{{\mathrm{SI}}_{\mathrm{post}}\mathrm{ of\ the\ spleen }}& \mathrm{LMR }= \frac{{\mathrm{SI}}_{\mathrm{post}}\mathrm{\ of\ the\ liver}}{{\mathrm{SI}}_{\mathrm{post}}\mathrm{\ of\ the\ muscle }}\end{array}\\ {}\end{array}$$

### ^99m^Tc-mebrofenin HBS

**Fig. 2:**
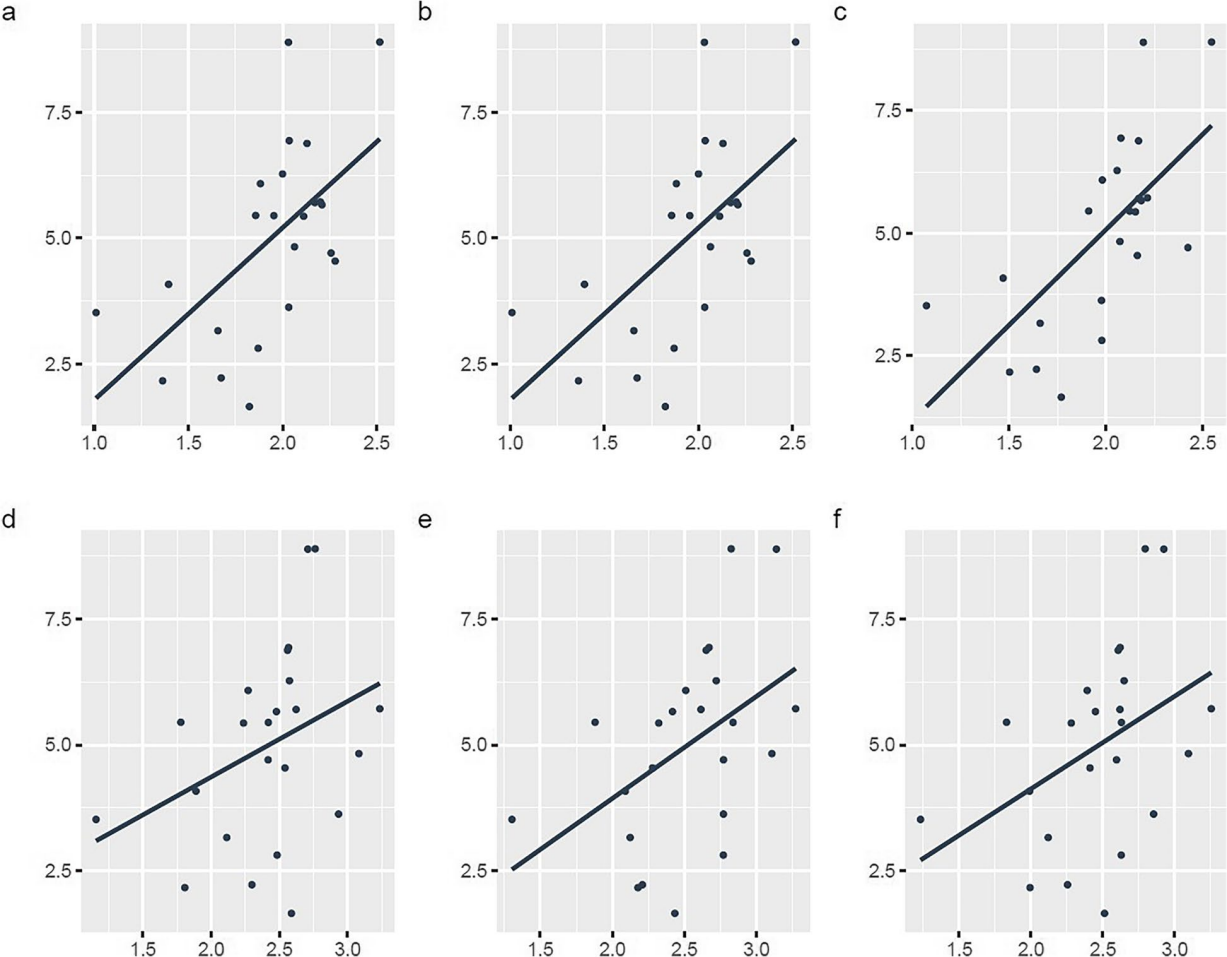
Correlation between mebrofenin uptake and MRI liver enhancement at baseline. Scatter plots with linear regression lines showing liver-to-spleen-ratio (LSR) *versus* mebrofenin uptake for right (**a**) and left (**b**) liver lobes and total liver (**c**). Liver-to-muscle-ratio (LMR) for each volume is presented in the same order (**d–f**)

**Fig. 3:**
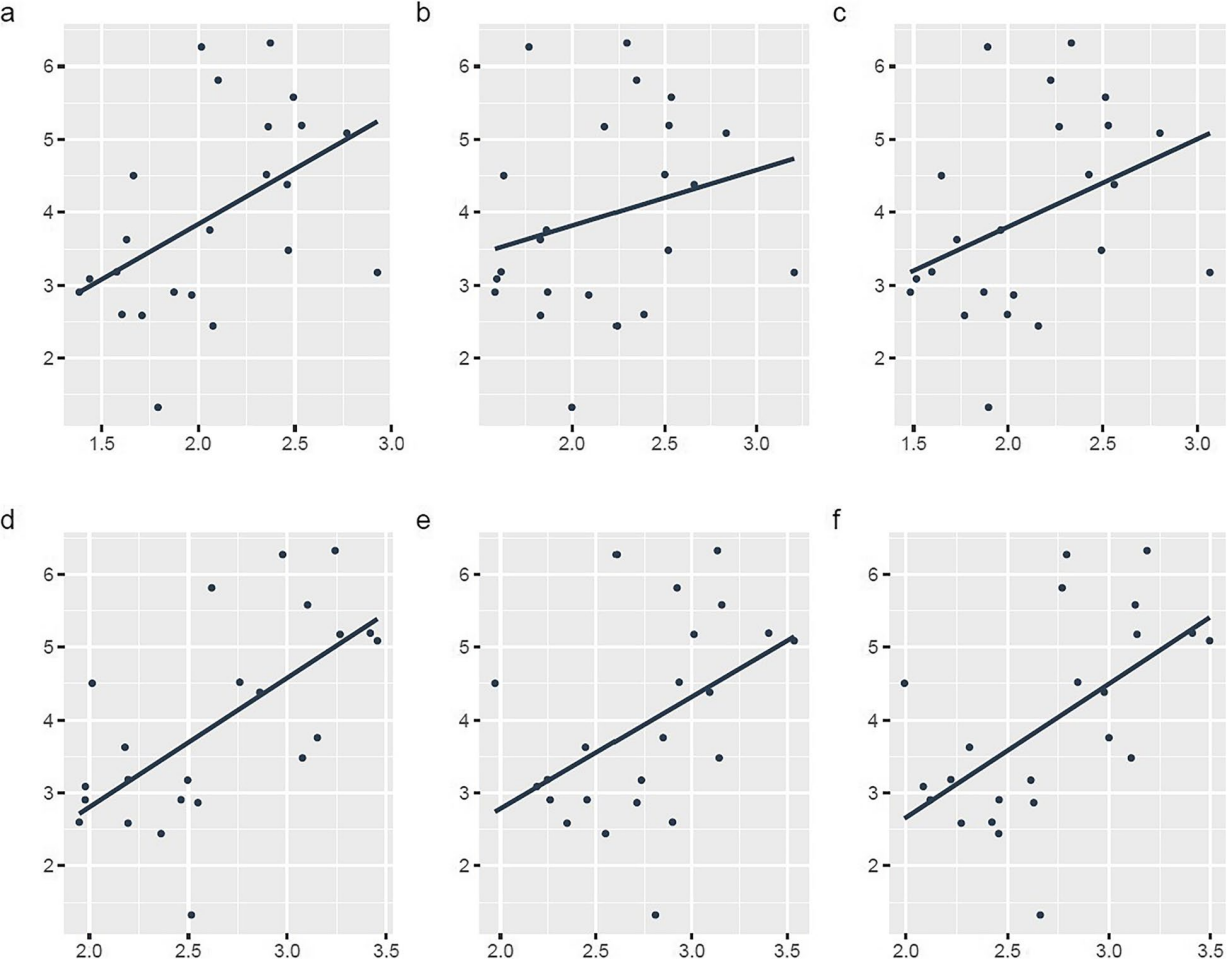
Correlation between mebrofenin uptake and MRI liver enhancement at follow-up. Scatter plots with linear regression lines showing liver-to-spleen-ratio (LSR) *versus* mebrofenin uptake for right (**a**) and left (**b**) liver lobes and total liver (**c**). Liver-to-muscle-ratio (LMR) for each volume is presented in the same order (**d–f**)

HBS was performed using a single-photon emission computed tomography/computed tomography (SPECT/CT) with ^99m^Tc-labelled-(2,4,6-trimethyl-3-bromo) iminodiacetic acid (^99m^Tc-mebrofenin, Bridatec; GE Healthcare, Buchler GmbH & Co. KG, Braunschweig, Germany). After intravenous administration of 150–370 MBq of ^99m^Tc-mebrofenin, dynamic planar imaging within 360 s, followed by rapid SPECT/CT imaging over 240 s, was used to determine the ^99m^Tc-mebrofenin uptake rate (Figs. 2 and 3). SPECT/CT imaging determined the counts within the liver and FLR volume. The percentage of the counts in the FLR in relation to the entire liver was then multiplied by the ^99m^Tc-mebrofenin uptake rate to obtain the actual metabolic function of the FLR. Calculations were performed to normalise the uptake rate to BSA and respective liver volumes (total, left, right) as described before [7]. Additionally, ICG (0.25 mg/kg) was administered intravenously to confirm the HBS findings, and ICG plasma disappearance rate and ICG retention at 15 min were measured non-invasively using a spectrophotometry clip attached to the patient’s index finger, with adjustments made for variations in cardiac output between baseline and follow-up measurements.

### Statistical analysis

All statistical analyses were performed using the R statistics software (Version 4.2.3). Continuous and ordinal variables are reported as median (interquartile range) if not otherwise specified, and categorical variables are reported as counts and percentages. Correlations were tested by calculating Pearson correlation coefficients after the normal distribution was checked by normal plots. To indicate statistical significance, *p*-values < 0.05 were considered.

## Results

Baseline characteristics of the analysed 23 patients are shown in Table 1. The median age was 66 (interquartile range 59–74) years, and most patients had metastatic colorectal cancer (11, 48%). The median time from baseline MRI to RE was 20 days (interquartile range 20–40), and the median time from RE to follow-up was 39 days (interquartile range 30–42). The average mebrofenin uptake normalised to body surface area in %/min/m^2^ dropped significantly between baseline and follow-up in the right liver lobe (5.0 *versus* 3.9, *p* = 0.023) whereas it increased in the left liver lobe after RE (1.3 *versus* 1.6, *p* = 0.048) (Table 2).

**Table 1:** Patient characteristics (*n* = 23)

Clinical data		
Age (min–max) (years)	66	(59–74)
Male/female	17/6	(73.9%/26.1%)
Liver cirrhosis	0	0%
Liver steatosis	5	21.7%
Previous chemotherapy	17	73.9%
Previous liver resection	4	17.4%
Primary tumour		
Colorectal cancer	11	47.8%
Cholangiocellular carcinoma	4	13.0%
Breast cancer	3	17.3%
Hepatocellular carcinoma	1	4.3%
Neuroendocrine tumour	1	4.3%
Renal cell carcinoma	1	4.3%
Oesophageal carcinoma	1	4.3%
Pancreatic ductal adenocarcinoma	1	4.3%
Mean (SD) ^90^Y dose to RLL (Gy/mL)	45.7	(7.5)

Data are absolute number and percentages unless differently indicated

*RLL* Right liver lobe, *SD* Standard deviation

**Table 2:** Hepatobiliary scintigraphy liver function

	Baseline	Follow-up	*p*-value
HBS total liver in %/min/m^2^ (SD)^a^	6.3 (2.5)	5.5 (1.8)	0.068
HBS right liver lobe in %/min/m^2^ (SD)^a^	5.0 (1.9)	3.9 (1.4)	0.023*
HBS left liver lobe in %/min/m^2^ (SD)^a^	1.3 (0.8)	1.6 (0.8)	0.048*

*HBS* Hepatobillary scintigraphy, *SD* Standard deviation

^a^HBS normalised to body surface area

^*^p-values with statistical significance

### Correlation of LSR with HBS

LSR at baseline and follow-up correlated significantly with mebrofenin uptake for TL (baseline, *r*^2^ = 0.635, *p* < 0.001; follow-up, *r*^2^ = 0.462, *p* = 0.013) and RLL (*r*^2^ = 0.597, *p* = 0.001; *r*^2^ = 0.483, *p* = 0.010). Regarding the LLL, a significant correlation was only seen at follow-up (*r*^2^ = 0.349, *p* = 0.051; *r*^2^ = 0.359, *p* = 0.046) (Table 3).

### Correlation of LMR with HBS

LMR at baseline and follow-up correlated significantly with mebrofenin uptake for TL (*r*^2^ = 0.473, *p* = 0.011; *r*^2^ = 0.679, *p* < 0.001), RLL (*r*^2^ = 0.353, *p* = 0.049; *r*^2^ = 0.644, *p* < 0.001), and LLL (*r*^2^ = 0.464, *p* = 0.013; *r*^2^ = 0.540, *p* = 0.004) (Table 3). Figures 2 and 3 show scatterplots.

**Table 3:** Correlation of MRI signal intensity with HBS

	Right liver lobe		Left liver lobe		Total liver	
	Correlation	*p*-value	Correlation	*p*-value	Correlation	*p*-value
*Baseline*						
LSR	0.597	0.001*	0.349	0.051	0.635	< 0.001*
LMR	0.353	0.049*	0.464	0.013*	0.473	0.011*
*Follow-up*						
LSR	0.483	0.010*	0.359	0.046*	0.462	0.013*
LMR	0.644	< 0.001*	0.540	0.004*	0.679	< 0.001*

*HBS* Hepatobillary scintigraphy, *LMR* Liver-to-muscle-ratio, *LSR* Liver-to-spleen-ratio, *MRI* Magnetic resonance imaging

^*^p-values with statistical significance

## Discussion

The utilisation of reliable tests to assess regional liver function represents a desirable advancement, as it has the potential to enhance the accuracy of predicting postoperative liver function. Global liver function tests may not adequately capture regional dysfunction or variations in functional and non-functional liver volume distribution, thereby limiting their ability to accurately predict postoperative liver function after hypertrophy-inducing procedures. For this, image-based methods are of utmost importance whereby HBS has emerged as a type of reference standard in the last few years [42–44].

Our study demonstrated a significant correlation between LSR and LMR derived from gadoxetic acid-enhanced MRI and HBS measurements after RE-based hypertrophy induction, suggesting that MRI can serve as a convenient alternative method for estimating FLR. Only the lack of correlation between baseline LSR on MRI and scintigraphy imaging of the LLL raises questions. This could potentially be attributed to artefacts introduced by the proximity of vital organs such as the heart, lungs, and stomach to the relatively small LLL, which might lead to inconsistent signal intensities affecting the accuracy of liver function assessment.

Gadoxetic acid-enhanced MRI has previously shown promising results in predicting post-hepatectomy liver failure, providing accurate anatomical information and reliable assessment of global and regional liver function in a single comprehensive examination [45–47]. Moreover, MRI-based liver function evaluation can be seamlessly integrated into routine clinical practice without the need for additional sequences, making it accessible in various medical centres. This accessibility allows patients residing far from specialised liver centres to be monitored locally, minimising the need for travel and facilitating optimal timing of resection. Additionally, the lack of ionising radiation exposure in MRI is advantageous, and gadoxetic acid-enhanced MRI can also contribute to the diagnosis and grading of liver fibrosis, providing valuable information for surgical planning in major hepatectomies [48]. In this regard, gadoxetic acid-enhanced MRI holds the potential to surpass ICG and HBS in the preoperative evaluation of liver function. Back in 2015, Geisel et al. [39] showed a good correlation between HBS and MRI in a cohort and postulated that they may equally be used to separately determine right and left liver lobe function.

To the best of our knowledge, this is the first study to show that this can also be applied in the setting after RE-based hypertrophy induction. It is imperative to acknowledge that the present study can be considered as a preliminary investigation, given the inherent limitations and the need for caution in establishing definitive cutoff values pertaining to the MRI-derived parameters. Existing data in this has predominantly originated from relatively small cohorts, thereby warranting prudence in generalising the findings. Nonetheless, it is not obligatory to employ a mandatory approach, as it is possible to conduct a patient-specific assessment by juxtaposing pre- and post-hypertrophy induction images, thereby providing important information to the FLR estimation. Furthermore, the measurements are susceptible to the influence of multiple factors, encompassing patient-specific variables like patient positioning, technical disparities in MRI machines, differences in imaging protocols, and inherent variability in the measurement techniques employed. However, it is important to recognise that valuable information is embedded in the image data, and we should strive to make use of it. It should also be noted that even with methods considered as standard of care, fluctuations in the predictive power of FLR estimates have been observed. For instance, previous studies have indicated a discrepancy between liver volume growth and liver function, with faster functional increase than volumetric increase after portal vein embolisation, but the opposite trend after the first stage of ALPPS [29, 42, 43, 49].

It must be mentioned that the early assessment timeframe of 6 weeks after RE is atypical as many studies have shown that the volume increases induced in the contralateral lobe by unilobar RE continue to increase for up to 12 months [50–52]. The 6-week follow-up period adopted in this study may not have fully captured the peak volume and potential function enhancements, possibly posing a constraint on our findings, which was already discussed in the main study manuscript [4]. Nevertheless, since this was the first prospective study of its kind and considering that a standard waiting period of 6 weeks post-portal vein embolisation as the competing method is typical in preparation for major hepatectomy, the decision to conduct a 6-week follow-up was considered suitable.

In addition to the aforementioned limitations, it should be noted that our study utilised only one reader. However, multiple ROIs were placed and accounted for. Additionally, the assessment was quantitative and not qualitative, contributing to a low potential for interreader reproducibility. Furthermore, it is worth noting that gadoxetic acid-enhanced MRI offers the possibility to derive other quantitative liver function parameters, including T1 relaxometry-based data, which have the potential to provide absolute values for the purpose of broader applicability and therefore may play a pivotal role in further exploring this subject [34]. Regrettably, these sequences entail a higher level of complexity and are not standard in routine liver MRI protocols. Consequently, due to the non-inclusion of these sequences in our study protocol, the exploration of such values was precluded in our analysis. However, it is important to underscore that one of our primary aims was to demonstrate the efficacy of utilising a standard MRI protocol to acquire information about increasing liver function without elongating or complicating the examination duration and protocol. Besides, LMR and LSR are among the most commonly used parameters and are robust in their calculation with high intra- and interobserver reproducibility, as previous studies have demonstrated [53]. In addition, it is essential to establish a correlation between preoperative MRI and mebrofenin HBS parameters, with actual postoperative outcomes, since the association between such surrogate measures and clinically significant events like posthepatectomy liver failure still requires further research with larger cohorts.

In conclusion, our study demonstrates that hepatobiliary phase MRI exhibits a correlation with ^99m^Tc-labelled mebrofenin uptake in HBS following RE-induced hypertrophy. This suggests that Gd-EOB-DTPA-enhanced MRI holds promise as an alternative to HBS, facilitating the assessment of contralateral liver lobe function enhancement after unilateral RE. This shift towards MRI has potential practical implications, as it offers a patient-friendly and environmentally conscious approach by eliminating the need for complex additional investigations.

## Data Availability

The data that support the findings of this study are available from the corresponding authors upon reasonable request.

## Abbreviations

ALPPS: Associating liver partition with portal vein ligation for staged hepatectomy
CT: Computed tomography
FLR: Future liver remnant
HBS: Hepatobiliary scintigraphy
ICG: Indocyanine green
LLL: Left liver lobe
LMR: Liver-to-muscle ratio
LSR: Liver-to-spleen ratio
MRI: Magnetic resonance imaging
RLL: Right liver lobe
ROI: Region of interest
SI: Signal intensity
SPECT: Single-photon emission computed tomography
TL: Total liver
